# Strontium isotope evidence for a highly mobile population on the Pamir Plateau 2500 years ago

**DOI:** 10.1038/srep35162

**Published:** 2016-10-20

**Authors:** Xueye Wang, Zihua Tang, Jing Wu, Xinhua Wu, Yiqun Wu, Xinying Zhou

**Affiliations:** 1 Key Laboratory of Cenozoic Geology and Environment, Institute of Geology and Geophysics, Chinese Academy of Sciences, Beijing 100029, China; 2 University of Chinese Academy of Sciences, Beijing 100049, China; 3 CAS Center for Excellence in Tibetan Plateau Earth Sciences, Beijing, China; 4 Institute of Archaeology, Chinese Academy of Social Sciences, Beijing 100071, China; 5 History Department of Humanities School, Xinjiang University, Urumqi 830046, China; 6 Key Laboratory of Vertebrate Evolution and Human Origin, Institute of Vertebrate Paleontology and Paleoanthropology, Chinese Academy of Sciences, Beijing 100044, China

## Abstract

Archeological researches have proposed arguments for human mobility and long-distance trading over the Eurasia before the Silk Roads. Here we utilize biologically available strontium isotope analysis to assess the extent of pre-Silk Road population movements and cultural communications across the Asian interior. From an early Iron Age cemetery (ca. 2500 yr B.P.) on the eastern Pamir Plateau, mean ^87^Sr/^86^Sr ratios from 34 individuals display considerable isotopic variability, and 10 individuals are distinguished as migrants based on the local strontium isotope range of 0.710296–0.710572 defined by 12 ovicaprine bones. Comparison of the proportion (10/34) with the regional census data completed in 1909 A.D. (3% non-locals) suggests a highly migratory behavior on the plateau 2500 years ago. Furthermore, exotic mortuary objects, such as silk fabrics from eastern China and angular harp originated from the Near East, clearly demonstrate an interaction between different cultures on the plateau before the establishment of the Silk Road.

Well-established civilizations over the eastern and western part of the Eurasian continent at least can be dated back to the Bronze Age. It is a long-standing viewpoint, however, that east-west communications crossing the continental interior had not burgeoned until the establishment of the Silk Roads at 114 B.C.^1^,^2^,^3^ (Fig. 1a). More recently, archaeological researches provide subtle hints for pre-Silk Road intercultural communications across inner Asia^4^. For instances, ancient Tarim inhabitants between 4000 and 3500 years before present (yr B.P.) had a diverse maternal ancestry originating from both the West and the East^5^,^6^, and a silk thread was found from a ca. 1000 B.C. mummy’s hair in ancient Egypt^7^,^8^. Although these discoveries have implied the existence of long-distance migration and trade, little information on the extent and intensity of interactions between the pre-Silk Road cultures is available yet.

Ancient cultural communications directly result from human migrations^9^, which can be effectively traced by strontium (Sr) isotopes in archaeological researches^10^. As a high-mass element, Sr shows no measurable isotope fractionation as Sr passes from weathered rocks up through food chain into mineralized tissues where Sr substitutes for Ca, and the ^87^Sr/^86^Sr values thus essentially depend on regional geologic background^11^,^12^. Animals typically consume plants over a wild area and hence generate homogenized strontium compositions, serving as reliable local values as human at the same location. In practice, the mean ^87^Sr/^86^Sr value with ±2 s.d. in archaeological animal bones is conventionally defined as the local range for charactering ancient migration^10^,^11^.

At the junction of East Asia, South Asia, and central Asia, the Pamir Plateau is both a communication corridor and a meeting place of various civilizations since the late Paleolithic Age^13^,^14^,^15^. We present here the first set of biologically available ^87^Sr/^86^Sr data from a 2500-year-old cemetery on the plateau to investigate the extent of pre-Silk Road human mobility around the region.

## Results

Results for all samples are listed in Tables 1 and 2, as well as illustrated in Fig. 2. The ^87^Sr/^86^Sr ratios measured in 12 ovicaprine bones range from 0.710303 and 0.710528 with a mean value of 0.710434 ± 0.000069 (1 s.d.). Accordingly, the local range for ^87^Sr/^86^Sr is 0.710296–0.710572.

Although the enamel samples are of higher variance for ^87^Sr/^86^Sr ratios, their mean value is 0.710428 ± 0.000217 (1 s.d.), almost identical to the mean value of ^87^Sr/^86^Sr ratios measured in the bone samples. There are 10 values falling out of the local range for the region (Fig. 2).

## Discussion

Twenty-four out of 34 individuals analyzed (70.6%) have ^87^Sr/^86^Sr ratios within the defined local range for the Jirzankal cemetery, suggesting they were locally born and raised. Meanwhile, the rest 10 individuals (29.4%) have ^87^Sr/^86^Sr ratios outside the local range, suggesting that they migrated to the cemetery from someplace else after childhood. These data indicate a highly mobile population settled on the Pamir Plateau around 2500 yr B.P. when compared to a historical population census data in the Kashgar where the cemetery affiliated. The census completed in 1909 A.D. demonstrated that only 3% non-locals, both from outside and inside Xinjiang Province, lived in the Kashgar Prefecture (17,215 migrants out of 599,578 populations^16^).

There is no gainsaying that mobile ovicaprine is not the best choice for defining local range for ^87^Sr/^86^Sr as human commensal species, particularly pigs. However, different from high-frequency co-occurrences of ovicaprine and human remains, we found rare bone fragments of eagle (n = 1), fox (n = 1), dog (n = 1) and rabbit (n = 2). Furthermore, isotope analysis shows that δ^15^N values of human bones (13.26 ± 0.55‰) are significantly higher than that of ovicaprine bones (8.33 ± 1.46‰, n = 13), indicating ovicaprine greatly contributed to human diet^17^. Considering its availability and high portion in diet, the ovicaprine bone with minor variance of ^87^Sr/^86^Sr values than human tooth enamel could be a preferred candidate for defining the local range.

Seasonal movement of herds might yield a broader range of ^87^Sr/^86^Sr than the accurate one. On the eastern Pamir Plateau, human beings mainly settle in low altitude river valley, ~3000 m a.s.l. and, in winter, they share the pastures with herds that graze on highlands more than 3500 m a.s.l. in summer. The highlands are geologically dominated by granitoid characterized by high ^87^Sr/^86^Sr values (0.82–1.2^18^,^19^), while the valley holds vast detrital deposits eroded from the granitoid with high ^87^Sr/^86^Sr values and Permian-Triassic marine carbonates typically of low values (0.7068–0.7080^20^,^21^). Consequently, the local range for ^87^Sr/^86^Sr of human values are enveloped by those of the herds, and mainly grouped in the lower part within the faunal range, as evidenced by our data distribution (Fig. 2). The non-local proportion (10/34) reported here is thus a minimum estimation for the immigrants.

Ten non-locals as identified by Sr isotope analysis come from seven graves. Among them, all three individuals from the grave M1 are non-locals with one below and two above the local range for Sr isotope ratios, and two out of four individuals from M14 are classified as migrants with Sr isotope ratios below the local range (Fig. 2). The non-local ^87^Sr/^86^Sr values falling both above (n = 7) and below (n = 3) the local range for the cemetery suggest the ancient inhabitants in the Pamir Plateau were derived from various places. Furthermore, the divergent ^87^Sr/^86^Sr values between enamel and ovicaprid bone from an identical grave, such as M17 and M18 (Tables 1 and 2), provide us a detailed immigrating scenario. The individuals moved onto the plateau after childhood and were buried with local ovicaprids after death, especially considering the significant differences of lifespan between human beings and ovicaprids.

**Figure 1:**
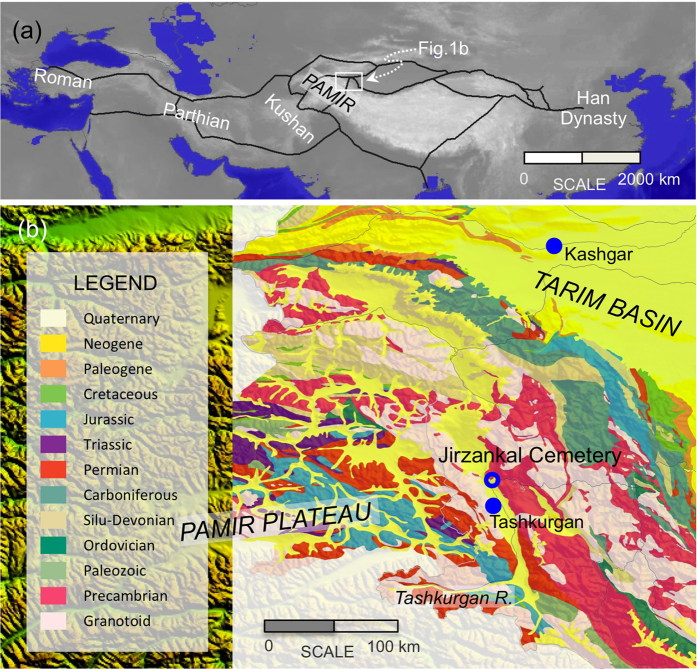
(**a**) Sketch map of the Silk Roads in the Han dynasty; (**b**) Simplified geologic map overlapping the topographic map of the eastern Pamir Plateau. Figures are designed and prepared by ZT using DIVA-GIS 7.5 (http://www.diva-gis.org).

**Figure 2:**
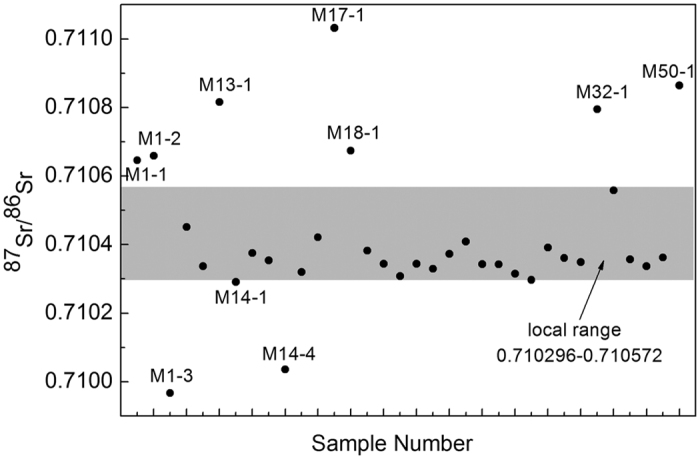
Scatter chart of ^87^Sr/^86^Sr ratios for human enamel with local range in gray.

Highly heterogeneous ^87^Sr/^86^Sr values clarify a complex geologic history on the regional (Fig. 1b) scales, and make it difficult to constrain the locations where migrants originated. Taking into consideration the harsh condition on the plateau, we here tentatively argue that the non-locals emigrated from locations beyond the Pamir Plateau. This argument was backed by the characteristic mortuary objects in the cemetery. During the two excavation seasons, 42 wooden objects and abundant wood/charcoal fragments were found from the cemetery and seven tree species have been identified^22^. Among them, junipers, silver berry, and willow can be found nearby on shady mountains slopes or on floodplains along the Tashkurgan River, while honeysuckle, syrian ash, birch, and poplar mainly develop in typical temperate climate. These introduced species account for the majority the wooden materials found in the cemetery^22^ and provide additional information for the migration distance of ancient inhabitants.

**Figure 3:**
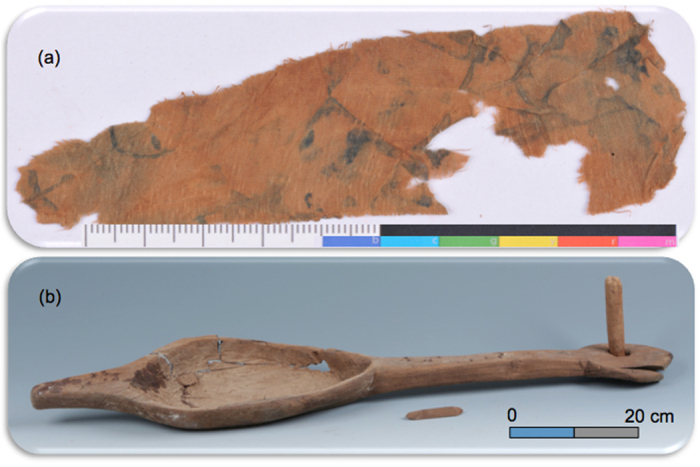
Typical mortuary goods in the cemetery. (**a**) fragments of silk fabric from the grave M1; (**b**) an angular harp from the grave M14.

There are some early Iron Age graves found within two nearby cemeteries, Xiangbaobao^23^ and Xiabandi^24^. These cemeteries, especially the Xiangbaobao cemetery, were slightly older than the Jirzankal cemetery and prevailed cremation, a strikingly contrast to the dominance of shaft burials in the Jirzankal cemetery presented here. The burial customs of the Jirzankal cemetery indicate some uniqueness of the study site. In our study cemetery, some typical mortuary goods accompanied with non-local individuals are ideal archives for cultural transmission. As mentioned above, the graves M1 and M14 hold three and two non-locals, respectively. In M1 the discoverers have found fragments of silk fabric (Fig. 3a). It is widely accepted that silk was first produced in eastern China around the fourth or fifth millennium B.C.^25^. After being closely guarded for generations, the culture of silkworm and the manufacturing technology of silk exported westward since the Han dynasty along the Silk Roads. According to official documents, the knowledge and technique reached the Tarim Basin by 50 A.D.^26^ and further India by 140 A.D.^27^. Beyond these regions, silk gradually symbolized the pre-modern China and bridged the empire to the western world.

Meanwhile, a set of wooden musical instrument was found in the M14 (Fig. 3b). It is made of ash^22^ and independently dated to 2450 ± 30 yr BP (Table 3, Beta-403048, 2710–2360 cal. yr B.P.), composing of a long-necked hollow trough that is assumed to a sound box, a rod perpendicular to the neck through a square hole assumed to a tuning contraption, and a thin stick to fasten the rob^28^. This instrument has been recognized as an angular harp, a kind of ancient stringed instrument originated in the Near East^29^. The earliest harps were arched, found in Sumer around 3000 B.C.^30^, and angular harps followed around 1900 B.C.^31^. Probably carried by nomadic tribes such as the Scyths, angular harps quickly penetrated the Central Asian steppe eastward during the first millennium B.C. and arrived at the Pamir area ca. 500 B.C., as presented here. Almost at the same time or slightly later, the harps appeared in Xinjiang, NW China^32^ and finally reached East Asia by 2^nd^ century B.C.^33^.

Silk is the symbol of agriculture civilization of eastern China, while harp is a typical musical instrument originated in the Near East. Their presences as mortuary goods on the Pamir Plateau strongly suggest a vigorous long-distance cross-culture communications ca. 2500 year ago.

Strontium isotopes provide a promising method of separating immigrants with ^87^Sr/^86^Sr ratios out of the local range. We use the ratios in dental enamel to distinguish migrant individuals for an early Iron Age cemetery (ca. 2500 yr B.P.) for the first time and our results confirm a highly mobile population on the Pamir Plateau. The coexistence of particular mortuary goods, such as silk fabrics originated from East China and angular harp from the Near East, strongly suggests far-reaching cultural interactions on the Pamir Plateau long before the opening of the Silk Roads.

### Study Site and Specimens

Jirzankal cemetery (75°12′11′′N, 37°50′54′′E) is located on the western bank of the Tashkurgan River, eastern Pamir Plateau (Fig. 1b). The region, 3050 meters above sea level, has a typical alpine climate with long winters and brief summers. The cemetery is approximately 10 km away from the Tashkurgan City to the south and 180 km away from Kashgar, the capital of the prefecture where the cemetery situated.

Thirty-nine graves were excavated in 2013–2014. A typical grave within the cemetery was a shaft chamber covered by encircling stones. The chambers with primary and secondary burials were dug into bedrock or fluvial gravel layer. During the two excavation seasons, at least 39 individual human corpses and two hundred mortuary goods were revealed. The mortuary goods included abundant pottery items, stone tools, wooden items, textiles, sporadic copper objects and ironware. AMS radiocarbon dating of various samples from nine graves, including five bones, six wood/charcoal fragments and one textile, jointly assign the cemetery to ca. 2500 yr B.P., corresponding to the early Iron Age (Table 3).

Bone samples from 12 ovicaprids (goat/sheep) and enamel samples from 34 individuals were analyzed for strontium isotope ratios. Depending on availability and preservation, mostly molars were selected with an exception of premolar (sample M25-3).

## Methods

Teeth were abraded with a carbide dental burr on a dental drill to remove any visible contamination from the crown and a sample (10–20 mg) was taken from the crown, while bone samples were ashed at 750 °C for 8 hours^10^,^34^. All samples were cleaned with deionized water and dissolved in Teflon-PFA vials using 2 mL of 0.2N HCl. Sr was separated from matrix using AG50W × 12 resin, a Sr-selective resin (200–400 mesh diameter) loaded into the tip of a 2 mL Teflon column. The whole procedure blanks for Sr were typically lower than 250 picograms. Isotopic ratios were measured on a Finnigan MAT262 thermal ionization mass spectrometer (TIMS) housed at the State Key Laboratory of Lithospheric Evolution at the Institute of Geology and Geophysics, Chinese Academy of Sciences (IGGCAS) using the internal ratio ^88^Sr/^86^Sr = 8.375209 to correct for mass fractionation^35^. All ^87^Sr/^86^Sr isotope ratios were normalized to a value of ^87^Sr/^86^Sr = 0.71025 for NBS-987 standard, which was run concurrently with samples and produced an average value of 0.710269 ± 0.000012 (2 s.d.) during this work (n = 9).

## Additional Information

**How to cite this article**: Wang, X. *et al.* Strontium isotope evidence for a highly mobile population on the Pamir Plateau 2500 years ago. *Sci. Rep.* **6**, 35162; doi: 10.1038/srep35162 (2016).

## Figures and Tables

**Table 1:** ^87^Sr/^86^Sr ratios for ovicaprine bones.

Sample No.	Sample	^87^Sr/^86^Sr
1	M10	0.710445
2	M11	0.710429
3	M16	0.710351
4	M17	0.710528
5	M18	0.710504
6	M22	0.710468
7	M23	0.710413
8	M25	0.710303
9	M28	0.710371
10	M29	0.710401
11	M30	0.710502
12	M46	0.71049

‘M*i*’ represents grave number.

**Table 2:** ^87^Sr/^86^Sr ratio for human tooth enamel.

Sample No.	Sample	Sex	FDI Notation	^87^Sr/^86^Sr
1	M1-1	Male	46	0.710646
2	M1-2	Female	46	0.710659
3	M1-3	?	26	0.709967
4	M11-1	Female	27	0.710451
5	M11-2	Female	47	0.710337
6	M13-1	Female	48	0.710816
7	M14-1	Male	48	0.710291
8	M14-2	Female	38	0.710375
9	M14-3	Male	37	0.710354
10	M14-4	Male	38	0.710036
11	M15-2	Female	37	0.71032
12	M16-1	Female	37	0.710421
13	M17-1	?	37	0.711032
14	M18-1	Female	47	0.710674
15	M23-1	Male	48	0.710382
16	M24-2	Male	47	0.710344
17	M24-3	Male	47	0.710308
18	M24-4	Male	46	0.710344
19	M25-1	Male	18	0.710329
20	M25-2	Male	37	0.710373
21	M25-3	Female	34	0.710409
22	M25-4	?	36	0.710343
23	M26-1	Female?	16	0.710342
24	M27-1	Female	47	0.710315
25	M30-1	Male?	46	0.710297
26	M30-2	Female	27	0.710391
27	M31-1	Female	47	0.710361
28	M31-2	Male	46	0.710349
29	M32-1	Male	16	0.710795
30	M32-2	Female	38	0.710558
31	M35-1	Male?	38	0.710357
32	M35-2	Female	37	0.710337
33	M46-1	?	46	0.710362
34	M50-1	Female?	17	0.710864

The sample is marked as M*i-j*, where ‘M*i*’ represents grave number and ‘*j*’ represents individual number in the grave. The element of tooth is coded with the FDI’s two-digit notation system.

**Table 3:** List of radiocarbon dates for the Jirzankal cemetery.

Grave	Material	Lab No.	δ^13^C (‰)	Conventional age (yr B.P.)	Calibrated age* (±2σ, cal. yr B.P.)
M1	textile/wool?	Beta-354583	−19.6	2560 ± 30	2750–2550
M1	wood	Beta-354584	−23.7	2510 ± 30	2740–2470
M10	bone/human	Beta-360538	−17.9	2450 ± 30	2710–2360
M11	bone/human	Beta-360540	−17.6	2390 ± 30	2650–2360
M12	bone/human	Beta-360543	−17.4	2390 ± 30	2650–2350
M14	wood	Beta-360547	−23.3	2370 ± 30	2460–2340
M14	wood/harp	Beta-403048	—	2450 ± 30	2710–2360
M14	wood/arrowhead	Beta-400296	−21.7	2570 ± 30	2750–2700**
M15	wood	Beta-400297	−22.3	2430 ± 30	2540–2355**
M25	bone/human	Beta-403044	−17.3	2440 ± 30	2705–2355
M35	wood	Beta-403051	−24.1	2410 ± 30	2685–2350
M50	wood	Beta-403053	−23.3	2490 ± 30	2730–2460

^*^Calibrated using the IntCal13 curve.

^**^Cited from ref. 22.
